# Use of next-generation amplicon sequencing to study *Blastocystis* genetic diversity in a rural human population from Mexico

**DOI:** 10.1186/s13071-019-3814-z

**Published:** 2019-11-27

**Authors:** Liliana Rojas-Velázquez, Jenny G. Maloney, Aleksey Molokin, Patricia Morán, Angélica Serrano-Vázquez, Enrique González, Horacio Pérez-Juárez, Cecilia Ximénez, Monica Santin

**Affiliations:** 1 Unidad de Investigación en Medicina Experimental, Facultad de Medicina, Universidad Nacional Autónoma de México (UNAM), Mexico City, Mexico; 2 Environmental Microbial and Food Safety Laboratory, Agricultural Research Service, United States Department of Agriculture, Beltsville, MD USA; 3 Unidad de Posgrado, Universidad Nacional Autónoma de México (UNAM), Mexico City, Mexico

**Keywords:** *Blastocystis*, Human, Mexico, Mixed infections, Next-generation sequencing, Risk factors, Subtypes

## Abstract

**Background:**

The intestinal parasite *Blastocystis* is found in humans and animals around the world. It is spread through the consumption of contaminated food and water and has been associated with a variety of intestinal symptoms. *Blastocystis* is one of the most common intestinal parasites in humans, yet its prevalence and distribution in humans in North America is not well characterized.

**Methods:**

Next-generation amplicon sequencing of a region of the *Blastocystis SSU* rRNA gene was applied to DNA extracted from fecal specimens obtained from 182 inhabitants of a rural population in Mexico to characterize *Blastocystis* prevalence, subtype distribution, and intra-host subtype diversity in humans.

**Results:**

Of the 182 samples tested in this study, 68.1% (124) contained one or more *Blastocystis* subtypes. Subtype 3 was the most common subtype observed and was found in 81.5% of the positive samples. Subtype 1, 16.9% of the positive samples, and subtype 2, 17.7% of the positive samples, were also found in this population. Mixed infections were observed in 13.7% of the positive samples. In this population, the odds of having *Blastocystis* increased in adulthood (> 15 years; OR: 1.72, *P* < 0.0001), and the odds of having subtype 1 increased in the presence of farm animals (OR: 1.51, *P* = 0.03). The odds of having subtype 1, subtype 2, or a mixed infection decreased in the presence of cement flooring (OR: − 1.61, *P* = 0.005; OR: − 1.14, *P* = 0.03; OR: − 1.48, *P* = 0.02) possibly indicating socioeconomic factors are involved in the risk of acquiring one of these subtypes.

**Conclusions:**

These data contribute to our understanding of the epidemiology of *Blastocystis* infection in humans and can be used to shape future studies which aim to better characterize the transmission pathways and health outcomes of *Blastocystis* infections.
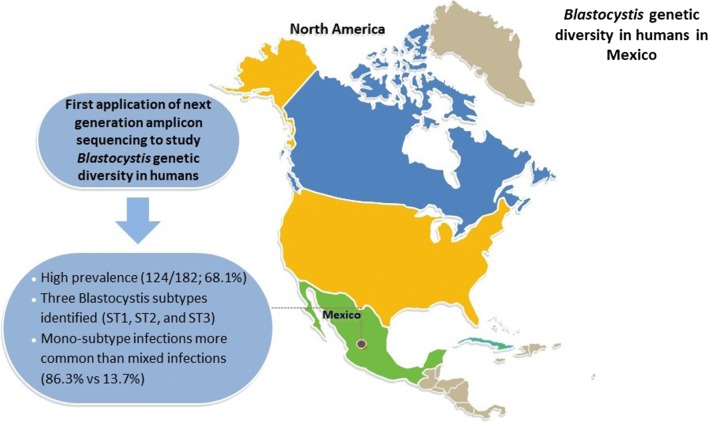

## Background

*Blastocystis* is a cosmopolitan enteric parasite found in humans and a wide range of animals across the world. Currently, *Blastocystis* is the most common intestinal parasite in humans in developing and developed countries [1]. However, the role of *Blastocystis* as a pathogen is still controversial, mainly because it is found in both patients suffering with intestinal symptomatology, such as diarrhea, flatulence, bloating or abdominal discomfort, as well as in healthy people [2]. *Blastocystis* has also been associated with irritable bowel syndrome and with cutaneous symptoms (urticaria) [2, 3]. *Blastocystis* is transmitted *via* the fecal-oral route either indirectly through ingestion of food or water contaminated with cysts or directly by contact with infected persons or animals [4].

A high degree of genetic diversity has been found among *Blastocystis* isolates based on nucleotide differences in the small subunit (*SSU*) of the ribosomal RNA (rRNA) gene. So far, at least 26 subtypes (STs) have been proposed [5–7]. Ten subtypes, ST1-ST9 and ST12, have been reported in humans, and of these subtypes all but ST9 are also found in other mammalian and avian hosts indicating the potential for zoonotic transmission [8, 9]. Of the ten subtypes reported in humans, ST1 to ST4 are most commonly found in humans worldwide [10]. *Blastocystis* in humans in North America is not well characterized at the molecular level. In fact, in the USA, only one molecular survey of *Blastocystis* has been performed in humans and found ST1, ST2 and ST3 were all present in humans from Colorado [11]. In Mexico, most molecular studies have been conducted in patient populations to understand the association between *Blastocystis* and irritable bowel syndrome and have reported ST1, ST2, ST3 and ST7 in humans [12, 13].

Molecular characterization to identify subtypes present in samples is critical to unravel *Blastocystis* epidemiology and to characterize subtype level differences in host specificity, transmission, public health significance, and pathogenicity. Mixed subtype infections are often overlooked in molecular studies of *Blastocystis*, and a better characterization of these infections is needed to fully understand the epidemiology of *Blastocystis*. Recently, it was demonstrated that next-generation amplicon sequencing is a powerful tool to investigate mixed infections and detect low abundance subtypes of *Blastocystis* [14]. The aim of the present study was to investigate *Blastocystis* in a rural population from Mexico using next-generation amplicon sequencing to better characterize *Blastocystis* prevalence, subtype distribution, and intra-host subtype diversity in humans and to evaluate potential association of socioeconomic factors with *Blastocystis* infection in this population.

## Methods

### Study population

One hundred and eighty-two volunteers living in the community of Xoxocotla, State of Morelos (Mexico) participated in the study conducted between May and November 2014 that included 86 males, 96 females, 66 children (≤ 15 years-old), and 116 adults (> 15 years-old) with age ranging from 2 to 51 years (median age of 23 years). Each participant provided three fecal samples that were collected on three consecutive days. The samples were maintained at 4 °C and transported to the laboratory in Mexico City. The sample size was calculated considering the total number of inhabitants in Xoxotla (21,074). The minimum required sample size was calculated to be 96 individuals based on an expected frequency of intestinal parasitic infection of 50%, the worst acceptable level was 10%, the confidence level was 95%, and the results were considered statistically significant when *P* < 0.05.

### DNA extraction

A similar volume of the three samples provided by each participant was combined and mixed thoroughly. Then, an aliquot of 250 mg from the mixture was used to extract genomic DNA using the QIAamp DNA Stool Mini Kit (Qiagen, Hilden, Germany) per manufacturer’s instructions. DNA was stored at − 20 °C until further molecular analysis.

### Molecular detection, NGS amplicon library preparation and bioinformatic analysis

Next-generation amplicon sequencing libraries were prepared as previously described [6]. Briefly, all samples were screened by PCR using primers ILMN_Blast505_532F and ILMN_Blast998_1017R. These primers amplify a region of the *SSU* rRNA gene and are identical to Blast505_532F/Blast998_1017R [15], with the exception of containing the Illumina overhang adapter sequences on the 5′-end. Final libraries were quantified using the Quant-iT dsDNA Broad-Range Assay Kit (Thermo Fisher Scientific, Waltham, MA, USA) on a SpectraMax iD5 (Molecular devices, San Jose, CA, USA) prior to normalization. A final pooled library concentration of 8 pM with 20% PhiX control was sequenced using Illumina MiSeq 600 cycle v3 chemistry (Illumina, San Diego, CA, USA). Paired end reads were processed and analyzed with an in-house pipeline that uses the BBTools package v38.22 [16], VSEARCH v2.8.0 [17], and BLAST+ 2.7.1. After removing singletons, clustering and the assignment of centroid sequences to operational taxonomic units (OTU) was performed within each sample at a 98% identity threshold. Only those OTUs with a minimum of 100 sequences were retained. All raw fastq files were deposited to the NCBI sequence read archive under the accession number PRJNA523857. The nucleotide sequences for unique OTUs obtained in this study have been deposited in GenBank under the accession numbers MK874780-MK874822.

### Sociodemographic variables

At the time of providing the samples, a questionnaire was administered to collect information on the following variables: age (child ≤ 15 years-old or adult > 15 years-old), gender (male or female), presence of symptoms (present or absent), type of flooring (dirt or cement), water source (city or other source), sewage disposal (in-house or other disposal), presence of animals (livestock, poultry, or companion), and presence of house pests (present or absent). The status of symptomatic was defined per the ROME III criteria commonly used by clinicians to classify gastrointestinal disorders.

### Data analysis

Logistic regression analysis was used to identify factors associated with *Blastocystis* infection. The following demographic and socioeconomic variables were included: age (child or adult), presence of symptoms defined as answering yes to one or more Rome III criteria (asymptomatic or symptomatic), type of flooring (dirt or cement), water source (city water or other source), sewage disposal (in-house or other source), presence of domestic animals (yes or no), presence of farm animals (yes or no), presence of chickens (yes or no), and presence of house pests (yes or no). Collected sociodemographic information for this population is presented in Table 1. *P*-values < 0.05 were considered statistically significant. Statistical analyses were performed using R version 3.5.1 (R Core Team, 2018).

**Table 1:** Sociodemographic variables studied by logistic regression analysis

Variable	*Blastocystis*			*Blastocystis* ST1			*Blastocystis* ST2			*Blastocystis* ST3			*Blastocystis* Mixed STs		
	Pos/Neg	*P*-value	Log odds	Pos/Neg	*P*-value	Log odds	Pos/Neg	*P*-value	Log odds	Pos/Neg	*P*-value	Log odds	Pos/Neg	*P*-value	Log odds
Age															
≤ 15 years	95/21	**< 0.0001**	**1.72**	14/103	0.9	− 0.09	19/97	**0.02**	**1.60**	78/38	**0.0003**	**1.36**	13/103	0.4	0.52
> 15 years	29/37			7/59			3/63			23/43			4/62		
Gender															
Male	54/33	0.07	0.66	9/78	0.7	0.23	12/75	0.5	− 0.36	45/42	0.2	0.39	10/77	0.3	− 0.62
Female	70/25			12/83			10/85			39/56			7/88		
Symptoms															
Asymptomatic	98/46	0.2	− 0.58	17/127	0.3	− 0.70	16/128	0.9	0.07	84/60	**0.04**	− **0.88**	16/128	0.08	− 2.00
Symptomatic	26/12			4/34			6/32			17/21			1/37		
Flooring															
Dirt	56/26	1	− 0.02	15/67	**0.005**	− **1.61**	15/67	**0.03**	− **1.14**	39/43	0.08	0.61	11/71	**0.02**	− **1.48**
Cement	68/32			6/94			7/93			62/38			6/94		
Water															
City	101/44	0.6	− 0.26	16/129	0.7	− 0.24	17/128	0.7	− 0.18	85/60	0.4	− 0.39	15/130	0.1	− 1.45
Other	23/14			5/32			5/32			16/21			2/35		
Sewer system															
In-house	26/12	0.9	0.05	2/36	0.1	1.73	4/34	0.6	0.42	22/16	0.9	− 0.04	2/36	0.1	1.42
Other	98/46			19/125			18/126			79/65			15/129		
Domestic animals															
Presence	75/30	0.4	0.68	14/91	0.2	2.19	16/89	0.2	2.39	56/49	0.9	− 0.14	9/96	0.2	2.08
Absence	49/28			7/70			6/71			45/32			8/69		
Farm animals															
Presence	39/12	0.2	0.57	10/41	**0.03**	**1.51**	8/43	1	− 0.03	28/23	0.8	0.13	6/45	0.3	0.77
Absence	85/46			11/120			14/117			73/58			11/120		
Poultry															
Presence	10/3	0.7	0.27	2/11	0.6	0.57	3/10	0.4	0.89	6/7	0.6	− 0.38	1/12	0.9	0.25
Absence	114/55			19/150			19/169			95/74			16/153		
House pests															
Presence	80/33	0.5	− 0.53	14/99	0.1	− 2.55	16/97	0.2	− 2.02	61/52	1	0	9/104	0.1	− 2.60
Absence	44/25			7/62			6/63			40/29			8/61		

*Abbreviations*: Pos/Neg, Positive/Negative

*P* < 0.05 are in bold

## Results

### *Blastocystis* prevalence

**Fig. 1:**
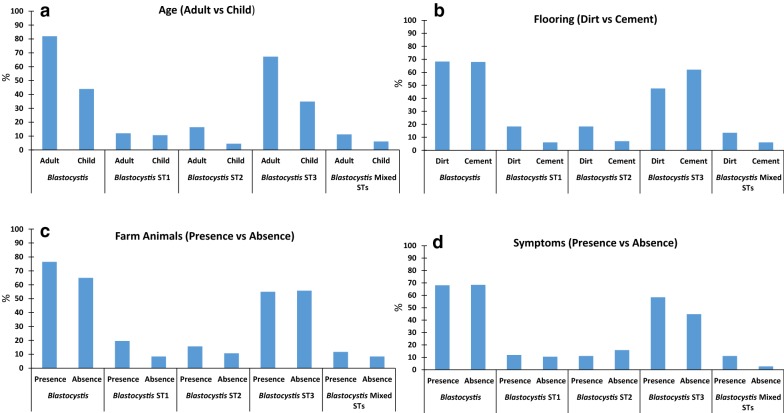
Prevalence of *Blastocystis* (any subtype or subtype combination), ST1, ST2, ST3, and mixed STs considering the variables: (**a**) age (adult *vs* child); (**b**) type of flooring (dirt *vs* cement); (**c**) presence of farm animals (presence *vs* absence); and (**d**) presence of symptomatology (presence *vs* absence)

Of the 182 samples screened 68.1% (124) were found to contain *Blastocystis* by PCR. A higher prevalence was observed in adults (81.9%; 95/116) than in children (43.9%; 29/66), and in females (62%; 54/87) than in males (74%; 70/95) (Table 1). A similar prevalence was found for asymptomatic (68%; 98/144) and symptomatic (68%; 26/38) participants (Table 1 and Fig. 1).

### *Blastocystis* subtypes identified using next-generation amplicon sequencing

All 124 PCR-positive samples were sequenced using the MiSeq platform. A total of 17,514,676 read pairs were generated from the samples included in this study with an average of 141,247 reads per sample. Following trimming, pair merging, and quality filtering there were a total of 4,968,142 merged reads. After chimera filtering 4,784,056 remained. Clustering yielded 176 *Blastocystis* OTUs across the 124 *Blastocystis*-positive samples of which 43 (24.4%) OTUs were unique (Table 2).

**Table 2:** Unique operational taxonomic units (OTUs) obtained for *Blastocystis* subtypes by next generation amplicon sequencing

ST	No. of unique OTUs per subtype	Unique OTU ID# (GenBank ID)	No. of samples containing OTU
ST1	15	1a (MK874787)	5
		1b (MK874795)	4
		1c (MK874813)	4
		1d (MK874786)	3
		1e (MK874810)	3
		1f (MK874789)	2
		1g (MK874797)	2
		1h (MK874807)	2
		1i (MK874796)	1
		1j (MK874798)	1
		1k (MK874802)	1
		1l (MK874816)	1
		1m (MK874817)	1
		1n (MK874819)	1
		1o (MK874822)	1
ST2	16	2a (MK874794)	7
		2b (MK874785)	6
		2c (MK874792)	5
		2d (MK874793)	4
		2e (MK874806)	4
		2f (MK874790)	3
		2g (MK874803)	3
		2h (MK874814)	2
		2i (MK874815)	2
		2j (MK874804)	1
		2k (MK874805)	1
		2l (MK874808)	1
		2m (MK874809)	1
		2n (MK874811)	1
		2o (MK874818)	1
		2p (MK874821)	1
ST3	12	3a (MK874780)	60
		3b (MK874781)	21
		3c (MK874782)	4
		3d (MK874783)	4
		3e (MK874784)	4
		3f (MK874801)	2
		3g (MK874788)	1
		3h (MK874791)	1
		3i (MK874799)	1
		3j (MK874800)	1
		3k (MK874812)	1
		3l (MK874820)	1

Three *Blastocystis* subtypes (ST1, ST2 and ST3) were detected in this study. Mono-subtype infections were more common than mixed infections (more than one subtype present in a single sample) representing 86.3% (*n* = 107) and 13.7% (*n* = 17) of the positive samples, respectively (Table 3; Additional file 1: Table S1). Subtype 3 was the most frequently observed subtype in this population and was found in 81.5% (*n* = 101) of the positive samples either as mono-infection (*n* = 84) or a mixed infection (*n* = 17) (Table 3). Subtypes 1 and 2 were observed in 16.9% (*n* = 21) and 17.7% (*n* = 22) positive samples, respectively, as either mono-infections (9 ST1 and 14 ST2) or mixed (12 ST1 and 8 ST2). A mix of ST1 and ST3 was the most common subtype combination and was found in 52.9% (*n* = 9) of the mixed infection samples. A mix of ST2 and ST3 was observed in 29.4% (*n* = 5) of mixed infections, and a mix of ST1, ST2 and ST3 was observed in 17.6% (*n* = 3) of the mixed infection samples. No ST1 and ST2 mixed infections were detected (Table 3).

**Table 3:** *Blastocystis* prevalence for each subtype in mono-infections and for the different subtype combinations in mixed subtype infections

	Mono subtype infections				Mixed subtype infections			
	ST1 only	ST2 only	ST3 only	Total mono-infections	ST1/ST3 mix	ST2/ST3 mix	ST1/ST2/ST3 mix	Total mixed infections
Total positive samples	9	14	84	107	9	5	3	17
Percentage of all samples	4.9	7.7	46.2	58.8	4.9	2.7	1.6	9.2
Percentage of positive samples	7.3	11.3	67.7	86.3	7.3	4.0	2.4	13.7
Percentage of mixed samples	na	na	na	na	52.9	29.4	17.6	na

*Abbreviations*: na, not applicable

### Intra-subtype variability

Forty-three unique OTUs were detected among the three *Blastocystis* subtypes present in this study. Subtype 1 and 2 had similar intra-subtype diversity in this study with 15 unique OTUs among 21 ST1-positive samples and 16 unique OTUs among the 22 ST2-positive samples (Table 2). Subtype 3 displayed the least intra-subtype diversity with only 12 unique OTUs among 101 ST3-positive samples. Samples frequently contained multiple unique OTUs of ST1 and ST2, and up to three unique OTUs of ST1 or ST2 were detected in individual samples (Table 3). However, multiple OTUs of ST3 were not observed in the same sample. Furthermore, while unique OTUs of ST1 and ST2 were relatively evenly distributed among individual samples, two unique OTUs of ST3 were dominant in this population and were observed in 81 of 101 *Blastocystis* ST3-positive samples (Table 2).

### Association between sociodemographic variables and presence of *Blastocystis*

Logistic regression analyses were performed to determine if any associations existed between *Blastocystis* infection and gender, adulthood, presence of symptoms, type of flooring, water source, sewage disposal, presence of domestic animals, presence of farm animals, presence of chickens, or presence of house pests (Table 1). Adulthood was the only variable with a statistically significant association with *Blastocystis* infection (any subtype or subtype combination) (Fig. 1a). The odds of having *Blastocystis* was greater in the adult category (OR: 1.72, 95% CI: 0.95–2.49, *P* < 0.0001), and adulthood also had a statistically significant association with being ST2- and ST3-positive when these outcomes were considered individually (OR: 1.6, 95% CI: 0.23–2.97, *P* = 0.02; OR: 1.36, 95% CI: 0.62–2.09, *P* = 0.0003) (Fig. 1a). Flooring type was also statistically significant for the outcomes of mixed STs, ST1- and ST2-positive (OR: − 1.48, 95% CI: − 2.70 to − 0.27, *P* = 0.02; OR: − 1.61, 95% CI: − 2.74 to − 0.48, *P* = 0.005; OR: − 1.14, 95% CI: − 2.17 to − 0.11, *P* = 0.03) (Fig. 1b). The odds of having mixed STs, ST1 or ST2 infection were all found to be lower if a sample came from a person living in a home with a cement floor. This effect was not observed for *Blastocystis* infection (any subtype or subtype combination) or ST3 infections. The presence of farm animals increased the odds of being ST1-positive (OR: 1.51, 95% CI: 0.18–2.84, *P* = 0.03) (Fig. 1c). Being symptomatic decreased the odds of being ST3 positive, although the association was weak (OR: − 0.88, 95% CI: − 1.73 to − 0.03, *P* = 0.04) (Fig. 1d). No other statistically significant associations between *Blastocystis* infection status or individual subtypes were found.

## Discussion

*Blastocystis* is a common intestinal parasite found in humans and many other animals, and its prevalence and subtype distribution have been described in human populations from around the world [10]. However, molecular studies of *Blastocystis* in North America remain lacking. This study describes the prevalence and subtypes of *Blastocystis* present in a rural population in Mexico for which data on socioeconomic and demographic variables was also collected. This study also represents the first application of next-generation amplicon sequencing (NGS) of the *Blastocystis SSU* rRNA gene to a human population to characterize intra-host subtype diversity.

Fecal samples from 182 humans ranging in age from 2 to 51 years from a single rural community in Mexico were screened for the presence of *Blastocystis* by PCR of the *SSU* rRNA gene follow by NGS to determine subtypes. One or more *Blastocystis* subtype was found in 68.1% of the samples. While this prevalence is on par with other studies of *Blastocystis* prevalence in rural populations from both the Americas and other regions of the world, it is on the higher end of the reported prevalence of *Blastocystis* in humans. Diagnostic methods used in different studies could account for prevalence differences. Indeed, a lower prevalence was reported for *Blastocystis* for this same study population using microscopy (59.9%;109/182) with molecular confirmation in only 66% of those 109 microscopy positives [18].

From the 124 positive samples in this study, subtypes 1, 2 and 3 were found. These three subtypes are frequently reported in humans in the Americas and around the world [10]. Subtype 3 was the dominant subtype in this population, and was observed in 81.5% of *Blastocystis-*positive samples and 55.5% of the study population. Subtypes 1 and 2 were observed much less frequently in 16.9% and 17.7% of the positive samples, respectively. These results are similar to observations of *Blastocystis* subtypes from a multi-country study in South America and a study in Brazil where ST3 was the most prevalent subtype found among *Blastocystis*-positive human samples followed by ST1 and ST2 [8, 19]. Both studies reported other subtypes in low numbers, ST4-ST8, ST12, and novel subtypes in the multi-country study and ST4, ST6 and ST8 in the study from Brazil. However, no other *Blastocystis* subtypes were observed in the samples in this study. Similarly, in a study in the USA that examined 50 family units (101 adults and 38 children/adolescents) from Colorado only subtypes ST1 (20%), ST2 (30%), and ST3 (50%) were identified [11]. In a survey of intestinal parasites in members of the Tapirapé ethnic group from the Brazilian Amazon region, only ST1, ST2 and ST3 were detected, but in their study ST1 was the most frequently identified subtype [20]. These differences between studies could be due to the geographical restrictions of some studies and may reflect population level or climatic influences on risk of infection with different *Blastocystis* subtypes.

The worldwide prevalence of *Blastocystis* mixed infections has been estimated to be 6% from previous studies on *Blastocystis* subtype diversity in humans [21]. This topic is not well explored, and only one study has directly addressed *Blastocystis* mixed subtype infections in humans [22]. By using a nested PCR assay that can identify subtypes 1 through 4, Scanlan et al. [22] demonstrated that 22% of *Blastocystis-*positive samples previously shown to contain a single subtype contained multiple *Blastocystis* subtypes. An advantage of NGS is its ability to assess intra-host subtype diversity [6]. The present study identified 17 mixed infections representing 13.7% of all *Blastocystis* infections in the population. Mixed subtype infections are underrepresented compared to expectations from subtype prevalence. However, mixed infections in our study were within the range of observations from other human studies [21, 22]. Combinations of ST1 + ST3, ST2 + ST3, and ST1 + ST2 + ST3 were all observed. ST3 was observed in all mixed *Blastocystis* infections, ST1 in 12 mixed infections, and ST2 in eight mixed infections. Although the population studied here was limited in its *Blastocystis* subtype diversity, these data support the use of NGS for exploring mixed subtype infections in humans.

Intra-subtype variability could play a role in understanding *Blastocystis* transmission and pathogenicity [6, 14, 23–25]. In the present study, intra-subtype variability varied widely between the subtypes. While ST1 and ST2 had high proportions of unique sequences, ST3 was surprisingly homogeneous. Unique sequences represented 71.4% of ST1 sequences and 72.7% of ST2 sequences, but only 11.9% of ST3 sequences were unique. Two OTUs of ST3 also dominated in this population representing 80.2% of all ST3 observations. This lower level of intra-subtype variability has been reported before for ST3 both in humans and cattle [6, 26]. There was also no within-host variation in ST3 despite multiple OTUs of ST1 or ST2 being frequently observed in the same host. The homogeneity of ST3 in this population may indicate that ST3 is highly endemic in this community and may be acquired from a common source or may pass more easily between humans than the other subtypes observed in this study.

In previous studies *Blastocystis* infection has been associated with factors such as age, animal contact, and sanitation practices [27–29]. To better understand what factors might influence *Blastocystis* infection risk in this population, demographic and socioeconomic data was collected and logistic regression analysis was used to determine if any associations exist between having *Blastocystis*, mixed STs, ST1, ST2 or ST3 infections and any of these factors. Because only two of the 43 unique OTUs detected in this study were found in more than 10 samples, statistical analyses attempting to link unique OTUs with risk factors were not performed.

In this population, the only factor associated with having *Blastocystis* was adulthood, with the odds of being *Blastocystis-*positive being greater in the adult (> 15 years-old) category (OR: 1.72, *P* ≤ 0.0001). In the present study, 116 of the 182 samples were adults (> 15 years-old) and 81.9% were *Blastocystis-*positive while 43.9% of the 66 children were *Blastocystis-*positive. Age has been indicated as a risk factor for *Blastocystis* in other studies, and human infants and young animals tend to have lower infection rates than adults [6, 29–31]. Adulthood was also associated with increased odds of having an ST2 or an ST3 infection (OR: 1.6, *P* = 0.02; OR: 1.36, *P* = 0.03), but this association was not observed for mixed or ST1 infections. These results could indicate that behavioral or physiological differences between adults and children may be important in determining infection risk for specific subtypes of *Blastocystis*.

Flooring material was significantly associated with mixed, ST1, and ST2 infections, and the odds of having one of these types of infections was decreased in homes with cement floors (OR: − 1.48, *P* = 0.02; OR: − 1.61, *P* = 0.005; OR: − 1.14, *P* = 0.03). This outcome may capture some socioeconomic effect such as improved hygiene not directly measured in this study as socioeconomic status has been associated with infection risk previously [30]. Having farm animals significantly increased the odds of having ST1 (OR: 1.51, *P* = 0.03). No other subtypes were associated with animals in this study. These results may indicate that zoonotic transmission of ST1 occurs in this population although data on the subtypes circulating in the animals owned or handled by ST1-positive individuals would be necessary to confirm this finding. Notably, artiodactyls such as pigs and cattle are the second most common host reported for ST1 after humans, further supporting the potential for zoonotic transmission of this subtype [10].

Being symptomatic, defined as answering yes to one or more Rome III criteria, was significantly associated with ST3 infection, although the association was negative (OR: − 0.88, *P* = 0.04). Being symptomatic decreased the odds of having ST3. This association while significant was not strong and caution should be taken in overinterpretation of this result as associations between subtypes and symptomatology is still unclear. Some studies have indicated that there is no association between diarrhea and ST1, ST2 or ST3, but ST4 is associated with diarrhea and irritable bowel syndrome [32–34]. However, ST1 and ST3 have been associated with intestinal symptoms in other studies [35, 36].

Subtype 1 has been found in drinking water in Thailand and river water in Nepal, and waterborne transmission of *Blastocystis* to humans was suspected in these studies [28]. In the present study, no statistically significant association was found between infection and water source indicating that transmission in this community may occur through other routes. No statistically significant associations were found between any infection outcomes and sewage disposal, presence of domestic animals, presence of chickens, or presence of house pests.

## Conclusions

This study provides important information about the epidemiology of *Blastocystis* and represents the first application of a *Blastocystis*-specific NGS protocol to study *Blastocystis* in humans. Although the study population described had a relatively homogenous *Blastocystis* subtype community, infection status and individual subtypes could still be linked to specific risk factors. More studies which aim to characterize mixed subtype infections and intra-subtype variation are needed to understand the transmission dynamics, epidemiology, and pathogenicity of *Blastocystis* in humans and animals. NGS provides a valuable tool for achieving this goal.

## Supplementary information

**Additional file 1: Table S1.** Blastocystis subtypes relative abundance in positive samples identified by next generation amplicon sequencing.

## Data Availability

All raw fastq files were deposited to the NCBI sequence read archive under the accession number PRJNA523857. The nucleotide sequences for unique OTUs obtained in this study have been deposited in the GenBank database under the accession numbers MK874780-MK874822.

## Abbreviations

PCR: polymerase chain reaction
*SSU* rRNA: small subunit of the ribosomal RNA
ST: subtype
OTU: operational taxonomic unit
