# Supplementary material for: Use of next-generation amplicon sequencing to study Blastocystis genetic diversity in a rural human population from Mexico
Source: Parasit Vectors. 2019 Nov 27;12:566. doi: 10.1186/s13071-019-3814-z (PMC6882168; doi:10.1186/s13071-019-3814-z)
Supplement: Supplementary file 1 — Additional file 1: Table S1. Blastocystis subtypes relative abundance in positive samples identified by next generation amplicon sequencing. [file 13071_2019_3814_MOESM1_ESM.docx]

**Additional file 1: Table S1.** *Blastocystis* subtypes relative abundance in positive samples identified by next generation amplicon sequencing.

| **Human ID** | **Subtype/s identified (%)** |
| --- | --- |
| 1 | ST3 (100) |
| 2 | ST2* (100) |
| 3 | ST3 (100) |
| 4 | ST2* (100) |
| 5 | ST3 (100) |
| 7 | ST3 (100) |
| 8 | ST2* (100) |
| 10 | ST3 (100) |
| 11 | ST3 (100) |
| 13 | ST3 (100) |
| 15 | ST3 (100) |
| 16 | ST3 (100) |
| 18 | ST3 (100) |
| 19 | ST3 (100) |
| 21 | ST3 (100) |
| 22 | ST1 (34.2), ST3 (65.8) |
| 23 | ST3 (100) |
| 25 | ST3 (100) |
| 26 | ST3 (100) |
| 29 | ST2 (1.2), ST3 (98.8) |
| 30 | ST3 (100) |
| 32 | ST2 (1.4), ST3 (98.6) |
| 33 | ST1 (20), ST2* (53.2), ST3 (26.9) |
| 34 | ST3 (100) |
| 35 | ST1*(100) |
| 36 | ST3 (100) |
| 39 | ST3 (100) |
| 40 | ST2* (100) |
| 41 | ST1* (32.4), ST3 (67.6%), |
| 42 | ST1 (99.5), ST3 (0.5) |
| 43 | ST2* (100) |
| 44 | ST1 (100) |
| 46 | ST1 (5.1), ST2* (75.5), ST3 (19.5%) |
| 47 | ST1* (99.8), ST3 (0.2) |
| 49 | ST1 (75.4), ST3 (24.6) |
| 50 | ST1*(99.1), ST3 (0.9) |
| 52 | ST3 (100) |
| 55 | ST1 (0.3), ST3 (99.7) |
| 57 | ST3 (100) |
| 58 | ST3 (100) |
| 60 | ST3 (100) |
| 61 | ST3 (100) |
| 64 | ST3 (100) |
| 65 | ST3 (100) |
| 66 | ST3 (100) |
| 68 | ST3 (100) |
| 69 | ST3 (100) |
| 70 | ST3 (100) |
| 71 | ST3 (100) |
| 73 | ST2* (100) |
| 74 | ST3 (100) |
| 76 | ST3 (100) |
| 78 | ST2* (100) |
| 79 | ST1* (100) |
| 80 | ST2* (100) |
| 82 | ST2* (100) |
| 83 | ST1 (100) |
| 84 | ST3 (100) |
| 85 | ST2 (99.1), ST3 (0.9%) |
| 86 | ST3 (100) |
| 87 | ST3 (100) |
| 88 | ST3 (100) |
| 89 | ST2 (100) |
| 91 | ST3 (100) |
| 92 | ST3 (100) |
| 93 | ST1 (100) |
| 94 | ST3 (100) |
| 95 | ST3 (100) |
| 97 | ST3 (100) |
| 98 | ST3 (100) |
| 99 | ST3 (100) |
| 100 | ST2 (7.1), ST3 (92.9) |
| 101 | ST3 (100) |
| 102 | ST3 (100) |
| 103 | ST3 (100) |
| 104 | ST3 (100) |
| 106 | ST3 (100) |
| 107 | ST3 (100) |
| 109 | ST3 (100) |
| 111 | ST3 (100) |
| 112 | ST3 (100) |
| 115 | ST1* (98.4), ST3 (1.6%) |
| 116 | ST3 (100) |
| 117 | ST3 (100) |
| 122 | ST3 (100) |
| 123 | ST3 (100) |
| 126 | ST3 (100) |
| 127 | ST3 (100) |
| 128 | ST3 (100) |
| 130 | ST3 (100) |
| 131 | ST3 (100) |
| 132 | ST3 (100) |
| 133 | ST3 (100) |
| 135 | ST3 (100) |
| 136 | ST3 (100) |
| 139 | ST3 (100) |
| 140 | ST3 (100) |
| 142 | ST1* (100) |
| 143 | ST3 (100) |
| 145 | ST3 (100) |
| 146 | ST3 (100) |
| 148 | ST3 (100) |
| 149 | ST3 (100) |
| 151 | ST2* (100) |
| 152 | ST3 (100) |
| 154 | ST3 (100) |
| 155 | ST3 (100) |
| 156 | ST3 (1.3), ST2 (98.7) |
| 157 | ST3 (100) |
| 158 | ST2* (100) |
| 159 | ST3 (100) |
| 160 | ST3 (100) |
| 162 | ST3 (100) |
| 163 | ST1* (100) |
| 164 | ST1 (20.1), ST2 (59.6), ST3 (20.3) |
| 165 | ST1* (100) |
| 170 | ST1 (100) |
| 173 | ST2* (100) |
| 174 | ST3 (100) |
| 176 | ST3 (100) |
| 177 | ST2* (100) |
| 178 | ST1 (83.4), ST3 (16.6) |
| 180 | ST3 (100) |
| 181 | ST3 (100) |
| *Denotes intra-subtype variability | |
